# MeJA-induced hairy roots in *Plumbago auriculata* L. by RNA-seq profiling and key synthase provided new insights into the sustainable production of plumbagin and saponins

**DOI:** 10.3389/fpls.2024.1411963

**Published:** 2024-07-12

**Authors:** Yirui Li, Zi-an Zhao, Ju Hu, Ting Lei, Qibing Chen, Jiani Li, Lijuan Yang, Di Hu, Suping Gao

**Affiliations:** ^1^ College of Landscape Architecture, Sichuan Agricultural University, Chengdu, China; ^2^ College of Biology and Pharmacy, Yulin Normal University, Yulin, China; ^3^ School of Fine Arts and Calligraphy, Sichuan Normal University, Chengdu, China

**Keywords:** *Plumbago auriculata* L., hairy root, MeJA, plumbagin, saponin, synthesis pathway

## Abstract

Naturally synthesized secondary metabolites in plants are considered an important source of drugs, food additives, etc. Among them, research on natural plant medicinal components and their synthesis mechanisms has always been of high concern. We identified a novel medicinal floral crop, *Plumbago auriculata* L., that can be treated with methyl jasmonate (MeJA) for the rapid or sustainable production of natural bioactives from hairy roots. In the study, we globally analyzed the changes in the accumulation of plumbagin and others in the hairy roots of *Plumbago auriculata* L. hairy roots (PAHR) 15834 in *P. auriculata* L. based on 100 μmol/L of MeJA treatment by RNA-seq profiling, and we found that there was a significant increase in the accumulation of plumbagin and saponin before 24 h. To explain the principle of co-accumulation, it showed that MeJA induced JA signaling and the shikimic acid pathway, and the methylvaleric acid (MVA) pathway was activated downstream subsequently by the Mfuzz and weighted gene co-expression analysis. Under the shared metabolic pathway, the high expression of *PAL3* and *HMGR* promoted the activity of the “gateway enzymes” phenylalanine ammonia lyase (PAL) and 3-hydroxy-3-methylglutaryl CoA reductase (HMGR), which respectively induced the high expression of key reaction enzyme genes, including chalcone synthase (CHS), isopentenyl diphosphate (IPP), and farnesyl pyrophosphate synthase (FPS), that led to the synthesis of plumbagin and saponin. We speculated that large amounts of ketones and/or aldehydes were formed under the action of these characteristic enzymes, ultimately achieving their co-accumulation through polyketone and high-level sugar and amino acid metabolism. The study results provided a theoretical basis for carrying out the factory refinement and biosynthesis of plumbagin and saponins and also provided new ideas for fully exploiting multifunctional agricultural crops and plants and developing new agricultural by-products.

## 1 Introduction

Human health issues have been a hot topic for centuries. In recent years, with the emergence of natural product drug research, the enormous resources of millions of plants and their contributions to human health have been discovered (Singh et al., 2017). Researchers hope to fully tap into their functions and maximize their socioeconomic benefits (Gong et al., 2020; Ji et al., 2020). The naturally synthesized secondary metabolites in plants are considered important sources of drugs, food additives, etc (Zhou et al., 2024). Even with the rapid advances in modern medicine, it is still necessary to use natural plant extracts to meet human health needs (Oguntibeju, 2018). The use of inducers to regulate the biosynthesis of secondary metabolites is considered an important method to significantly increase the content of target metabolites, and it is also an effective means to maintain the natural authenticity of products (Sohn et al., 2022). Therefore, it is an important foundational work for production and use in healthcare, including solving the problem of low efficiency in the synthesis of secondary metabolites in plants and increasing the content of medicinal components through biotechnology for production, which is increasingly being sought after by scholars (Xu et al., 2017; Rogowska et al., 2022).

The application of exogenous jasmonates (JAs) has been fully recognized for their ability to trigger plant growth and development (Mao et al., 2023; Zhou et al., 2024). It has been proven to be a key signal in plants that plays an adaptive role in resisting abiotic stress, and it is also a major promoter in enhancing secondary metabolites such as phenols, terpenoids, and flavonoids (Zhou et al., 2024). MeJA can regulate gene transcription and upregulate the expression of enzymes related to the synthesis of biometabolites, thereby promoting the accumulation of key secondary metabolites (Mao et al., 2023; Moghadam et al., 2024; Zhang et al., 2024b). Meanwhile, it has been proven to stimulate the biosynthesis of secondary metabolites in plants with nearly four times higher potency (Shuang and Hong, 2020), such as the accumulation of saponins and total flavonoids, in a manner that is evident in hairy root cultures (Ali et al., 2014; Rogowska et al., 2022). Its main advantage is that MeJA, as a stress hormone, is a prominent stress hormone related to plant survival under stress conditions, and it could directly target the JA signaling pathway (Gunjegaonkar and Shanmugarajan, 2019). By stimulating the differential expression of transcription factors, MeJA regulated biosynthesis-related genes and promoted the biosynthesis of terpenes and phenylpropanoids (Yao et al., 2021). Therefore, these studies provided new ideas regarding the use of exogenous MeJA and the application of biotechnology for identifying candidate genes and understanding the potential biosynthesis and regulatory mechanisms of secondary metabolites (Mao et al., 2023).

**Figure 1:**
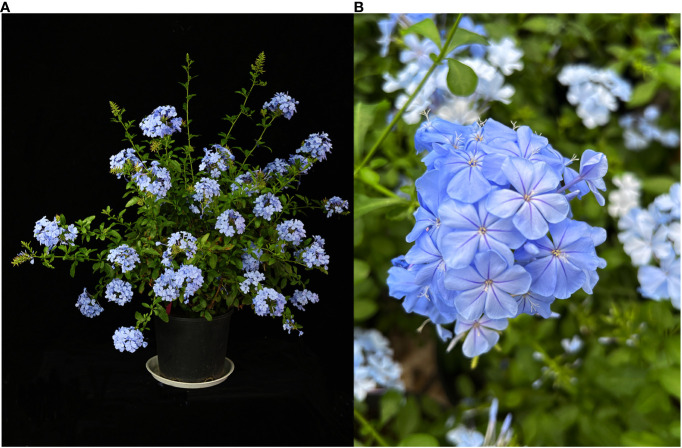
Ornamental traits in the horticulture of *P. auriculata*. **(A)** Ornamental traits during the flowering period of potted *P. auriculata* and **(B)** the inflorescence of *P. auriculata*.

*Plumbago auriculata* L. (*P. auriculata*) (**Figure 1**) is one of the representative plants of the Plumbaginaceae, *Plumbago*, which is capable of synthesizing the secondary metabolite plumbagin (5-hydroxy-2-methyl-1,4-naphthoquinone). Its plumbagin content is accumulated in large quantities in the roots or hairy roots and participates in important physiological metabolic processes (Han et al., 2002; Widhalm and Rhodes, 2016; Zhao et al., 2023), and it is studied to determine its anticancer, antidiabetic, anti-inflammatory, and antiradiation effects, among others (Checker et al., 2019; Wang et al., 2019c; Zhang et al., 2021; Liang et al., 2023; Shu et al., 2023). Of even greater interest is the great potential of the substance to function as a suppressor of many human and agricultural pathogens (Singh et al., 2017). In this study, we found that MeJA stimulated a large increase in plumbagin in the hairy roots of *P. auriculata*, which would provide an exciting research idea for expanding biopesticide inhibitors.

Although the biosynthetic pathways of quinones have been conjectured (Widhalm and Rhodes, 2016), the tissue-specific nature of the accumulation of such substances and their derivatives (Jadhav et al., 2014) posed a difficulty in their study, resulting in little information about their biosynthetic genes, intermediate formation, and regulation. Three ideas on the mechanism of their biosynthesis have been proposed in the existing studies: firstly, the biosynthesis of quinones was stimulated and controlled by auxin (Kumar et al., 2023), MeJA, ethylene (Yazaki et al., 1997), etc.; secondly, quinone biosynthesis was a process to stimulate transcriptional regulation of a large number of metabolic genes to regulate the synthetic reaction enzymes (Jiao et al., 2016; Vasav et al., 2020); and thirdly, phenylalanine was a precursor for the synthesis of quinones, and MeJA could act as a non-biological signal to regulate the expression of *PAL* in plants; thus, it achieved the synthesis of quinones through encoding phenylalanine ammonia-lyase (PAL) (Nopo-Olazabal et al., 2014; Widhalm and Rhodes, 2016). Based on the above, the involvement of a large number of reactive enzymes in the synthesis of plumbagin has been reported in *Plumbago indica* L. (*P. indica*) and *Plumbago zeylanica* L. (*P. zeylanica*) (Springob et al., 2006; Vasav et al., 2020). However, it is regrettable that there are no reports on the pathway of quinone biosynthesis due to inducer stimulation of the hairy roots, and there is a gap in the research on *P. auriculata*. Therefore, it has become an important and challenging issue to explore the biosynthetic pathways and regulatory mechanisms of plumbagin in a novel medicinal floral crop of *P. auriculata* and to promote its accumulation.

In addition to inducing the production of quinones, MeJA also induces other secondary metabolites (Sun et al., 2010). For example, MeJA stimulated the production of saponins in plants, which had similar medicinal properties to plumbagin (Seki et al., 2015; Guo et al., 2021; Chen et al., 2023). The principle is that MeJA acts as an inducer of the bioactive compound saponin by supplying the rate-limiting enzyme, 3-hydroxy-3-methylglutaryl CoA reductase (HMGR), through the terpene precursor isopentenyl diphosphate (IPP), which activates the HMGR activity and regulates the accumulation of the saponin substance (Pollier et al., 2013), and this phenomenon has also been verified in hairy roots (Kochan et al., 2019). Recent studies have found that the synthesis or accumulation of total plant saponins may first come from the contribution of farnesyl pyrophosphate synthase (FPS), thus demonstrating that HMGR and FPS are the key enzymes affecting saponin synthesis (Xu et al., 2018), and current studies have focused on the saponin synthesis pathway as well as on the methylvaleric acid (MVA) pathway (Lu et al., 2022) or the 2-C-methyl-D-rhodoseric acid 4-phosphate in the plastid (MEP) pathway (Zheng et al., 2021). In the present study, we were surprised to find that the hairy roots of *P. auriculata* stimulated by MeJA also promoted the large accumulation of saponins in addition to the efficient accumulation of plumbagin, a phenomenon not previously reported. Based on this interesting phenomenon, we were prompted to explore in-depth the key role played by those reactive enzymes and synthetic genes in the co-accumulation of plumbagin and saponin. How do they regulate the biosynthetic pathways of the two substances? Unfortunately, the answer was not given in previous reports.

In our previous study, we screened 100 μmol/L of MeJA as the optimal inducer treatment concentration using the hairy roots of *P. auriculata* established in a previous stage (Zhao et al., 2023). Under this concentration, the induced content of plumbagin produced was 1.56 times higher than that of the control. Based on this phenomenon, we conducted the study and analysis of RNA-seq and key metabolite accumulation in hairy roots. We aimed to explore the transcriptional activity and accumulation pathways that affect the synthesis of bioactive components such as plumbagin and also to clarify the key role of synthetic precursor substances in their future industrial production.

In conclusion, these results provided a theoretical basis and new insights for the comprehensive exploitation of the compound value of *P. auriculata*, as well as the biorefinement, development, and processing of functional compounds.

## 2 Materials and methods

### 2.1 Plant materials and treatments

The experimental materials were the hairy roots (PAHR 15834) of *P. auriculata* (Zhao et al., 2023). These were induced to form after 30 days using a culture of sterile leaves of *P. auriculata* by *Agrobacterium rhizogene*-mediated genetic transformation (ATCC15834), which was purchased from the American Type Culture Collection (ATCC), Rockville, Maryland, USA.

PAHR 15834, consisting of 600 bottles of monoclonal materials, was cultivated (**Supplementary Figure S1**). From these, 540 hairy roots, which had the same growth trend, were selected and placed in 100 mL of 1/2 MS liquid medium containing 10 μmol/L, 50 μmol/L, and 100 μmol/L of MeJA, respectively. The PAHR 15834 cultured in a medium supplemented with 95% ethanol in the same amount as MeJA was used as the control, with 135 replicates per treatment (45 replicates with 1 biological replicate, set 3 times). The incubation conditions were 25°C ± 2°C, 120 rpm, and constant temperature shock incubation in total darkness for 40 days. The samples were taken at 0 h, 4 h, 8 h, 16 h, 24 h, 48 h, 72 h, 5 days, 10 days, 15 days, 20 days, 25 days, 30 days, 35 days, and 40 days, respectively, where 0 h was set up as the control. The collected samples were repeatedly shaken and rinsed with ddH_2_O to remove excess culture medium. After cleaning, they were frozen in liquid nitrogen and stored at −80°C.

### 2.2 Plumbagin content in the hairy roots by HPLC

The study was carried out to determine the content of plumbagin in hairy roots using a modified HPLC method. Appropriate samples of PAHR 15834 at 0 h, 4 h, 8 h, 16 h, 24 h, 48 h, 72 h, 5 days, 10 days, 15 days, 20 days, 25 days, 30 days, 35 days, and 40 days were taken and dried at 65°C for 24 h and then adjusted to 45°C to dry to a constant weight. The dried samples were thoroughly ground and passed through a 40-mesh sieve, and the sieved powder was accurately weighed to 0.5 g and set aside.

To optimize our method based on the experimental method of Zhao et al. (2023), 25 mL of methanol (HPLC, purity ≥ 99.9%) was added to the samples by vortexing and shaking for 30 s and then ultrasonically extracted at room temperature for 30 min. The extracts were collected and filtered using 0.22-μm micropore filters, and the extracted liquid was placed in the dark at 4°C for backup. For testing (used Essentia LC-16, Shimadzu Hong Kong, China)) into 1.5-mL Agilent vials for content determination on the Agilent Technologies 1260 Infinity II liquid chromatography system and separated at a C18 column (Shim-pack GLS C18 4.6 × 250 mm, 5 μm) with a flow rate of 1 mL·min^−1^ and 40°C column temperature. The mobile phase A was acetonitrile, and liquid B was ultrapure water (using a 0.45-μm microporous filter membrane before use). The gradient elution steps were as follows: 40% A, 60% B, 0 min; 75% A, 25% B, 21 min; 90% A, 10% B, 23 min; and 90% A, 10% B, 30 min, at a flow rate of 1.0 mL·min^−1^, with a detection wavelength of 254 nm, separated at a column temperature of 40°C with a sensitivity of 0.16 AUFS, and an injection volume of 20 μL in all cases, with retention. The sensitivity was 0.16 AUFS, the injection volume was 20 μL, and the retention time was 21 min. A linear regression equation *y* = 150.3*x* − 126.3 (*R*² = 0.9999) was constructed for quantitative analysis using the standard of plumbagin (5-hydroxy-2-methyl-1, 4-naphthoquinone, CAS: 481–42-5, Sigma-Aldrich, Saint Louis, MO, purity ≥ 98%). At the same time, the optimal MeJA treatment concentration was screened according to the results of plumbagin content, and RNA-seq and other physiological indices were analyzed under the optimal treatment.

### 2.3 Analysis of soluble sugar and soluble protein content

Based on the method of Fan et al. (2022), we used approximately 0.1 g of the sample, added it to 1 mL of distilled water, and ground it into a homogenate. Then, it was poured into a centrifuge tube, sealed, and boiled in a water bath for 10 min. After cooling, the sample was centrifuged at 8,500 rpm at room temperature for 10 min. The supernatant was placed into a 10-mL test tube and diluted to 10 mL with deionized water. The test solution was prepared by following the instructions (ADS-WTDX039–50, Plant soluble sugar content detection kit, Jiangsu Aidisheng Biological Technology Co., Ltd. Jiangsu, China), and it was added to the sample and mixed well, placed in a water bath at 95°C for 10 min, and cooled to room temperature. The 200-μL liquid was transferred to a micro cuvette or a 96-well plate, and the absorbance values of the blank tube and the assay tube were read at 620 nm, respectively, Δ*A* = *A*_assay_ − *A*_blank_. The standard curve was established as *y* = 0.3154*x* − 0.0023 (*R* ^2^ = 0.9996). Based on the standard curve, the soluble sugar content was calculated.

Approximately 0.1 g of the sample was prepared, added to 1 mL of extraction solution, homogenized in an ice bath, and centrifuged at 10,000 rpm at 4°C for 10 min, and the supernatant was taken. The test solution was prepared by following the instructions of the manufacturer [ADS-F-SP001, Soluble Protein Content (BCA) Kit, (Jiangsu Aidisheng Biological Technology Co., Ltd. Jiangsu, China)]. The liquid was added to the sample, mixed well, and placed at 60°C for 30 min. The other steps were the same as for the soluble sugar content. Absorbances were read at 562 nm. The standard curve was established as *y* = 0.3154*x* − 0.0023 (*R* ^2^ = 0.9996). Based on the standard curve, the soluble protein content was calculated.

### 2.4 RNA preparation, transcriptome sequencing, library construction, and gene annotation

PAHR 15834 was treated with 100 μmol/L of MeJA, which was taken at 0 h (N), 8 h (T1), 16 h (T2), and 24 h (T3) for high-throughput sequencing analysis. Each treatment consisted of three biological replicates. After treatment, the hairy roots were washed and dried with distilled water, quickly frozen in liquid nitrogen, and then stored at −80°C until tested.

The extraction of total RNA from the tissue samples was done using the RNAprep Pure Plant Plus kit (Tiangen, Beijing, China) (Li et al., 2020a). RNA degradation and contamination were monitored by 1% agarose gel electrophoresis. RNA purity was assayed using NanoDrop 2000. RIN was detected using an Agilent 2100 RNA 6000 Nano Kit. The NEBNext^®^ Ultra™ RNA Library Preparation Kit was used for the library construction (Li et al., 2022). The libraries were assayed with Qubit 2.0 and Agilent 2100 systems. The transcriptome sequencing was based on SBS technology using Illumina HiSeq™ 4000 for raw reads (Fan et al., 2022). The above steps were completed by Gene Denovo Co., Ltd. (Beijing, China).

High-quality clean reads were obtained by removing splice sequences, read lengths containing an adaptor, read lengths with N ratios greater than 10%, and low-quality data (the number of bases with a quality value of *Q* ≤20 accounted for more than 40% of the entire read length). The clean data of each sample were assembled from scratch using the short read and long assembly software Trinity (version 2.0.6) in order to obtain the Unigene library. The functional gene annotation of Unigene sequences obtained from splice assembly by BLAST software was based on one or more of the following databases: NR, Swiss-Prot, GO, KOG, and eggNOG (Li et al., 2022). The homology results of Unigene in KEGG (Kyoto Encyclopedia of Genes and Genomes) were obtained using KOBAS 2.0. The functional annotation information for Unigene used the HMMER software against the Pfam database. The GO functional annotation information was obtained using Blast2GO software, the KOG functional classification statistics were performed on predicted genes (Shi et al., 2023), and the KEGG annotation was performed on Unigene to obtain pathway information (Cun et al., 2024).

### 2.5 Gene structure sequencing and gene expression analysis

Prediction of Unigene coding region sequences and their corresponding amino acid sequences was made using the TransDecoder (version r2014070464) software. Bowtie was used to compare the reads obtained from the sequencing of each sample with the Unigene library. Expression levels were evaluated in combination with RSEM, and FPKM values were utilized to indicate the expression abundance of the corresponding Unigene. Differential expression analysis was performed using EBSeq to obtain the set of differentially expressed genes between the two differently treated samples. The Benjamini–Hochberg method (*q*-value) was used to correct the significant *p*-value obtained from the original hypothesis test during the differential expression analysis, and the corrected *p*-value (i.e., FDR) was ultimately used as a key indicator for the screening of differentially expressed genes. Genes with an FDR <0.05 and |log2(FC)| ≥1 according to DESeq2 were considered differentially expressed genes (DEGs) (Liu et al., 2022).

### 2.6 Analysis of the content of total saponins and total flavonoids

Dried samples of PAHR 15834 taken at 0 h, 4 h, 8 h, 16 h, 24 h, and 48 h, weighing 0.05 g, were analyzed using the Total Saponins Content Assay Kit [ADS-400-F, Total saponin content test box, (Jiangsu Aidisheng Biological Technology Co., Ltd. Jiangsu, China)] for extraction and detection. One milliliter of extraction solution was added and ultrasonically extracted for 1 h, centrifuged for 10 min at 8,500 rpm at 25°C, and the total saponin content at 589 nm was analyzed (Uematsu et al., 2019).

Dried samples (0.1 g) of PAHR 15834 were taken at 0 h, 4 h, 8 h, 16 h, 24 h, and 48 h, and 1.5 mL of 60% ethanol was added and extracted by shaking at 60°C for 2 h. The centrifugation was carried out for 10 min, and the supernatant was taken at 25°C at 12,000 rpm. The sample was fixed with 60% ethanol to 1.5 mL, and the total flavonoid content [used ADS-W-KY007–48, Total Flavonoids (TF) Kit, Jiangsu Aidisheng Biological Technology Co., Ltd.] was determined at 510 nm (Huang et al., 2014).

### 2.7 Analysis of PAL, HMGR, C4H, 4CL, CHS, and FPS enzyme activities

The general enzyme activity assays served as our references (Rosler et al., 1997; Fan et al., 2022; Li et al., 2023a). The PAHR 15834 samples were taken at 0 h, 4 h, 8 h, 16 h, 24 h, and 48 h for enzyme activity assay. The PAL activity was assayed using MM-0899O2 Plant phenylalanine ammonia lyase (PAL) ELISA Research Kit (Jiangsu Meimian Industrial Co., Ltd. Jiangsu, China); C4H was assayed using MM-0942O2 Plant cinnamate-4-hydroxylase (C4H) ELISA Research Kit (Jiangsu Meimian Industrial Co., Ltd. Jiangsu, China); HMGR activity was assayed using MM-1929O2 Plant 3-hydroxy-3-methylglutaryl coenzyme A reductase (HMGR) ELISA Research Kit (Jiangsu Meimian Industrial Co., Ltd. Jiangsu, China); 4CL activity was assayed using MM-0945O2 Plant 4-coumaric acid coenzyme A ligase (4CL) ELISA Research Kit (Jiangsu Meimian Industrial Co., Ltd. Jiangsu, China); CHS activity was assayed using MM-2162O2 Plant chalcone synthase (CHS) ELISA Research Kit (Jiangsu Meimian Industrial Co., Ltd. Jiangsu, China); and FPS activity was assayed using MM-1522O2 Plant farnesyl pyrophosphate synthase (FPS) ELISA Research Kit (Jiangsu Meimian Industrial Co., Ltd. Jiangsu, China).

### 2.8 RT-qPCR expression level analysis of the key DEGs

Total RNA was extracted from the roots using the RNAprep Pure Plant Plus Kit (Tiangen, Beijing, China) and SteadyPure plant RNA Extraction Kit (Accurate Biotechnology (Hunan, China) Co., Ltd.), and residual genomic DNA was removed during the extraction process. The RNA concentration and quality were assayed with Thermo Scientific ND One, and the RNA integrity was assayed by 1.0% agarose gel electrophoresis. Reverse transcription of 1,000 ng of RNA into 20 μL of system cDNA was performed using the Evo M-MLV RT Mix Kit with gDNA Clean for qPCR Ver.2 (Accurate Biotechnology (Hunan, China) Co., Ltd.). ddH_2_O was used to dilute the cDNA to 30 μL for qPCR.

To verify the reliability of the RNA-seq data, RT-qPCR validation analysis was performed on randomly selected candidate genes that were contained in Unigene0000019, Unigene0012859, Unigene002300, Unigene0028761, Unigene0030931, Unigene0030968, and Unigene0039043. The RT-qPCR expression level analysis was used to identify candidate genes of interest on the KEGG synthesis pathway, including Unigene0015669 (*PAL3*), Unigene0002103 (*HMGR*), Unigene0033249 (*4CL1*), Unigene0031514 (*FPS2*), Unigene0033266 (*F26G*), Unigene0003471 (*IPP2*), and Unigene0035269 (*CHS2*). Primer 5.0 was used for the primer design, and *ACTIN* was used as the internal reference gene. All primers were obtained from Biotechnology Co., Ltd. (Shanghai, China, https://www.sangon.com), and the primer information is shown in **Supplementary Table S1**.

For RT-qPCR, the SYBR Green Pro Taq HS qPCR Kit (Accurate Biotechnology (Hunan) Co., Ltd.) was used. The 10-µL reaction system contained 5 µL of 2X SYBR Green Pro Taq HS Premix, 0.2 µL of forward primer (10 µmol/L), 0.2 µL of reverse primer (10 µmol/L), 1 µL of cDNA, and 3.6 µL of sterile water. Based on the study of Xu et al. (2018), we optimized the qPCR procedure as follows: 95°C, 3 min; 95°C, 15 s; 60°C, 30 s; the above steps were cycled 40 times, 65°C, 15 s; 95°C, 5 s. The Bio-Rad CFX96™ detection system was used. All reactions were repeated three times. The amplification products were detected by agarose gel electrophoresis and RT-qPCR solubility curves to verify the specificity of the primers. The relative expression levels of DEGs were calculated using the 2^−ΔΔCT^ method.

### 2.9 Data analysis

All data were statistically analyzed using SPSS 22.0, and a one-way ANOVA was used to determine the significant differences (*P* < 0.05). Pearson’s correlation analysis was conducted using Origin 2021. Excel 2016 was used for data sorting. GraphPad Prism 9, R language, TBtools, and Origin 2021 were used to graph the results. All data were subjected to not less than three biological replicates, and the analysis was expressed as *s* ± *x* (standard error, SE).

## 3 Results and analysis

### 3.1 Effect of MeJA treatment on the accumulation of plumbagin in hairy roots

**Figure 2:**
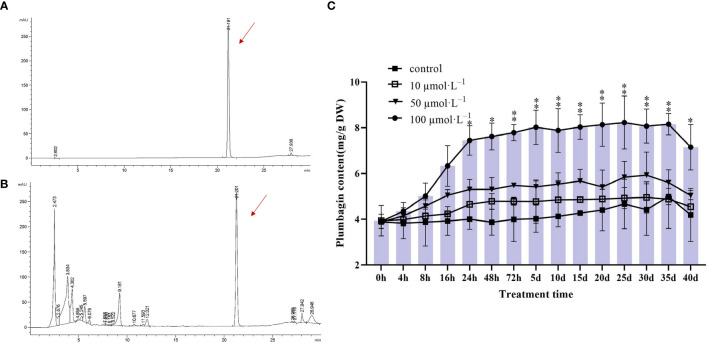
Plot of plumbagin content and trend of changes in PAHR 15834 at different times of MeJA treatment. **(A)** Peak diagram of plumbagin standard; **(B)** peak map of plant samples (one of the randomized samples is used as an example); and **(C)** bar graphs indicate the cumulative content of plumbagin at each time point, and line graphs indicate the trend of content accumulation of plumbagin in hairy roots over time under different MeJA treatments. Error bars indicate ± SE (*n* = 3). Asterisks indicate significant differences between different groups (*P < 0.05, **P < 0.01).

To investigate the dynamic changes of MeJA-induced accumulation of plumbagin in hairy roots, PAHR 15834 was dynamically sampled for content assay after 40 days of incubation. It was known from the study that both the PAHR 15834 extract and plumbagin standard had signals at 21 min, indicating that they belonged to the same substance and were both plumbagin (**Figures 2A, B**). Based on the above conditions, the samples at each stage were examined and calculated, showing that MeJA-treated PAHR 15834 had the best effect on stimulating the accumulation of plumbagin at 100 μmol/L of MeJA treatment (**Figure 2C**) compared with the control. At this concentration, the accumulation of plumbagin increased significantly to 7.44 mg/g in dry weight (DW) at 24 h, which was 1.91 times higher than that of the control group. For the next 2 days (48 h) to 35 days, plumbagin still accumulated, but the rising trend leveled off and reached a peak of 8.24 mg/g DW at 25 days, which was only an increase of 0.79 mg/g DW; plumbagin was decreased to 7.15 mg/g DW at 40 days, which was lower than the amount of plumbagin accumulation at 24 h. Therefore, MeJA treatment was effective in promoting the accumulation of plumbagin in hairy roots. Because of the consideration of production efficiency and cost, we experimentally only analyzed the samples at 0 h (control), 4 h, 8 h, 16 h, 24 h, and 48 h under 100 μmol/L of MeJA treatment.

### 3.2 Effect of MeJA treatment on soluble sugar and soluble protein content in hairy roots

**Figure 3:**
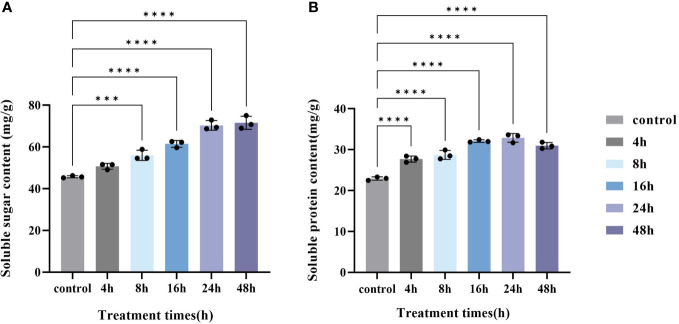
Changes in soluble sugar content and soluble protein content in PAHR 15834 at each sampling time after MeJA treatment. **(A)** Soluble sugar content and **(B)** Soluble protein content. Error bars indicate ± SE (*n* = 3). Asterisks indicate significant differences between different groups (***P < 0.001, ****P < 0.0001).

The soluble sugar and soluble protein contents were determined in PAHR 15834 at different treatment times (**Figure 3**). The soluble sugar content increased with the increase in MeJA treatment time (**Figure 3A**), and compared with the control, the soluble content increased significantly at 8 h, 16 h, 24 h, and 48 h (*P* < 0.05). The content at 48 h could be 25.86 mg/g higher than the control. The soluble protein content in PAHR 15834 showed an increasing and then a decreasing trend with the time of MeJA treatment (**Figure 3B**) and reached the extreme value of 22.93 mg/g at 24 h. Compared with the control, the soluble protein content in PAHR 15834 was significantly increased in all treatments (*P* < 0.05), which could be improved by 9.96 mg/g, 1.4 times the control. It indicated that MeJA could promote soluble sugar and soluble protein accumulation in PAHR 15834.

### 3.3 Global characterization of transcriptome data after RNA-seq analysis

The four treatment points of 0 h (control), 8 h, 16 h, and 24 h were selected for transcriptome sequencing. The total RNA quality of each extracted sample was examined, and the RNA concentration, OD value, RIN value, and 28S/18S ratio of each sample met the sequencing requirements before sequencing. After sequencing was completed, a total of 82.44 Gb of raw read lengths were obtained, and 77.33 Gb of clean read lengths were filtered. The GC content (%) of the 12 libraries ranged from 46.78% to 50.02%, with clean read lengths of 18.0 Gb or more for each sample and a Q30 of 92.57% or more (**Supplementary Table S2**). A total of 549,625,354 raw reads and 528,890,566 clean reads were obtained, of which clean reads for all samples were above 96%, fitness reads were above 0.5%, and low-quality reads were less than 3.5%. The assembly obtained 46,221 Unigenes, of which the GC percentage was 42.085 and the average length was 1,348 bp. These results indicated that the accuracy and quality of the sequencing data were highly amenable to further bioinformatics analysis.

### 3.4 Differential gene expression analysis reveals candidate genes involved in the biosynthesis of plumbagin and saponins

#### 3.4.1 Differential gene statistics

**Figure 4:**
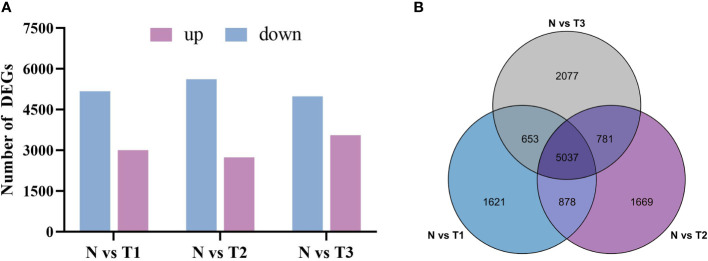
Statistical analysis of transcript differential genes. **(A)** The statistical plot of the number of up- and downregulated expressions of DEGs in the three treatment groups compared with the control group. **(B)** The Venn plot analysis of the three treatment groups compared with the control group.

**Figure 5:**
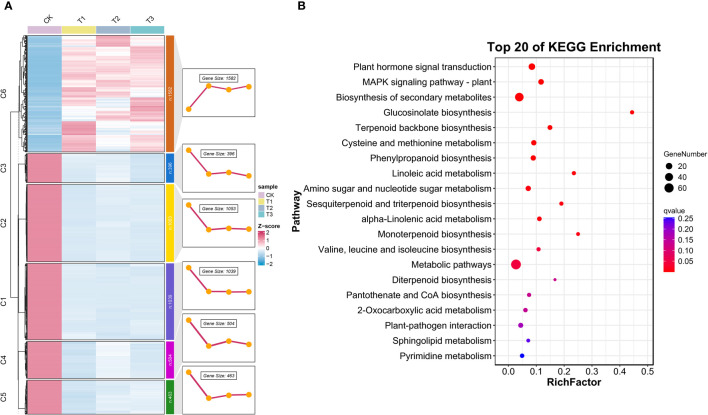
The Mfuzz and KEGG enrichment analyses in 5,073 DEGs. **(A)** Cluster analysis plot of the expression patterns of the six modules of a total of 5,073 DEGs identified based on the Mfuzz package in R. **(B)** KEGG-enriched analysis in 1,582 DEGs.

The number of differentially expressed genes between treatments and controls was counted using histograms with *P <*0.05 and log2|FC| ≥1 as screening conditions (**Figure 4A**). The set of differentially expressed genes common or unique to each analyzed combination was screened by a Venn plot (**Figure 4B**). As shown in **Figure 5A**, a total of 8,189 DEGs were screened in N vs. T1, with 3,008 upregulated genes and 5,181 downregulated genes. There were 2,741 upregulated genes and 5,624 downregulated genes identified in N vs. T2. The number of upregulated and downregulated genes in N vs. T2 were 2,741 and 5,624, respectively. In the 8,548 DEGs of N vs. T3, there were 3,650 upregulated genes and 4,988 downregulated genes. There were 5,037 identical differentially expressed genes in the three treatment groups compared with the control (**Figure 4B**), with 1,621, 1,669, and 2,077 DEGs specific to T1, T2, and T3, respectively. To better focus on the candidate genes that consistently affect plumbagin accumulation after MeJA treatment, we analyzed the 5,037 DEGs that were co-differentially expressed.

#### 3.4.2 Analysis based on Mfuzz gene expression patterns

Based on the results of the Venn plot analysis, to define the spatiotemporal expression characteristics of the transcriptome dataset, we used Mfuzz to perform cluster analysis of the 5,037 DEGs that were co-intersected in the 12 samples, classified them into six clusters, and demonstrated the gene expression trend of each cluster (**Figure 5A**). Notably, the expression trend of cluster 6 (C6) was different from the other clusters with an upward trend, and we hypothesized that it is essential and critical for the accumulation of the secondary metabolites. Finally, we targeted 1,582 genes in C6 and analyzed the KEGG pathways of the derived DEGs (**Figure 5B**), which were enriched in 15 KEGG pathways (RichFactor ≥0.1). The most notable pathways were “Phenylpropanoid biosynthesis,” “Cysteine and methionine metabolism,” “Terpenoid backbone biosynthesis,” “Pantothenate and CoA biosynthesis,” “Valine, leucine, and isoleucine biosynthesis,” etc., which proved our conjecture.

#### 3.4.3 Correlation analysis between co-expression networks of genes, physiological parameters, and DEGs

**Figure 6:**
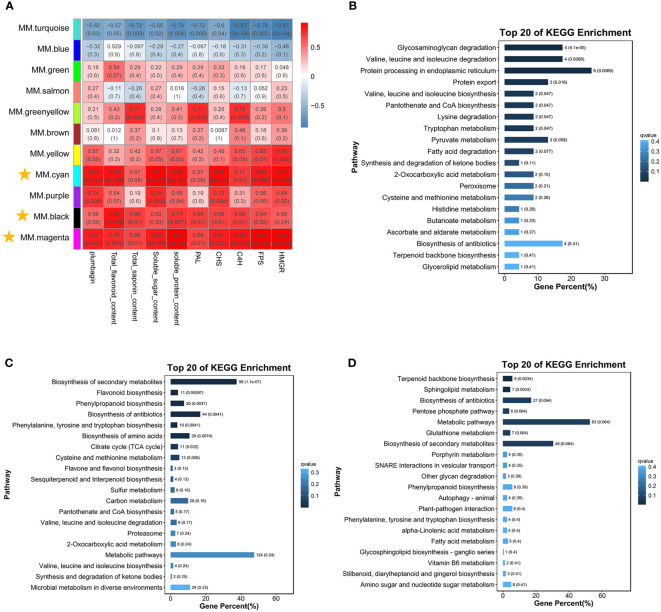
Correlation analysis between co-expression networks of genes, physiological parameters, and DEGs (Liu et al., 2022). **(A)** Co-expression networks were constructed using the WGCNA (v1.47) package in R. Gene expression values were imported into the WGCNA to construct co-expression modules using the automatic network construction function blockwiseModules with default settings. **(B–D)** These were the KEGG-enriched pathway maps of DEGs in the MM.cyan, MM.black, and MM.magenta modules, respectively.

Weighted correlation network analysis (WGCNA) is an analytical method for finding co-expressed gene modules and exploring the association of specific traits or phenotypes with gene networks (**Figure 6**). We tried to use this approach to gain a preliminary understanding of the key factors involved in the biosynthesis of saponins and plumbagin. The results of WGCNA were shown as 11 modules, and the correlation between modules and traits is shown in **Figure 6A**. Using *R* ^2^ ≥0.5 for both modules and phenotypic characteristics, almost all *P <*0.05, and consistent with the expression pattern of C6 gene expression as the screening criteria, we finally localized the three modules, MM.cyan (100 DEGs), MM.black (1,244 DEGs), and MM.magenta (894 DEGs), all of which had a high positive correlation.

The KEGG-enriched pathways of DEGs in these three modules were further analyzed, and in the MM.cyan module (**Figure 6B**), these DEGs were enriched (*P* < 0.05) in eight pathways, such as “Glycosaminoglycan degradation,” “Valine, leucine and isoleucine degradation,” “ Protein processing in endoplasmic reticulum,” and “Protein export”. In the MM.black module (**Figure 6C**), the DEGs were enriched in seven pathways, including “Biosynthesis of secondary metabolites,” “Flavonoid biosynthesis,” “Phenylpropanoid biosynthesis,” “Biosynthesis of antibiotics,” and “Phenylalanine, tyrosine, and tryptophan biosynthesis,” and seven other pathways were enriched (*P* < 0.05). The DEGs in the MM.magenta module (**Figure 6D**) were mainly enriched in “Terpenoid backbone biosynthesis” and “Sphingolipid metabolism” (*P* < 0.05). Therefore, we suggested that the genes in the MM.cyan module might mainly exercise the metabolite synthesis environment and energy consumption functions, and the genes in the MM.black and MM.magenta modules were related to metabolite biosynthesis.

#### 3.4.4 GO/KEGG enrichment analysis of DEGs based on WGCNA and Mfuzz

**Figure 7:**
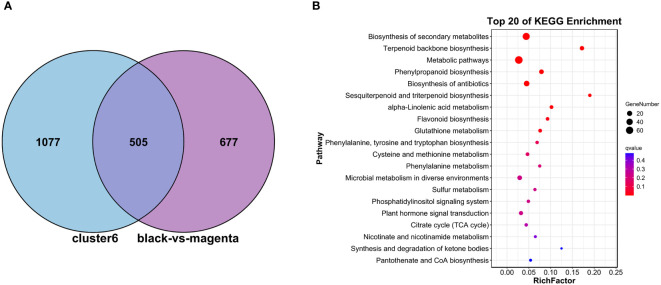
GO/KEGG analysis of DEGs shared between the WGCNA- and Mfuzz-screened modules. **(A)** Venn plot analysis of DEGs in cluster 6, MM.black, and MM.magenta. **(B)** KEGG analysis based on the 505 DEGs generated by the intersection in **(A)**.

**Figure 8:**
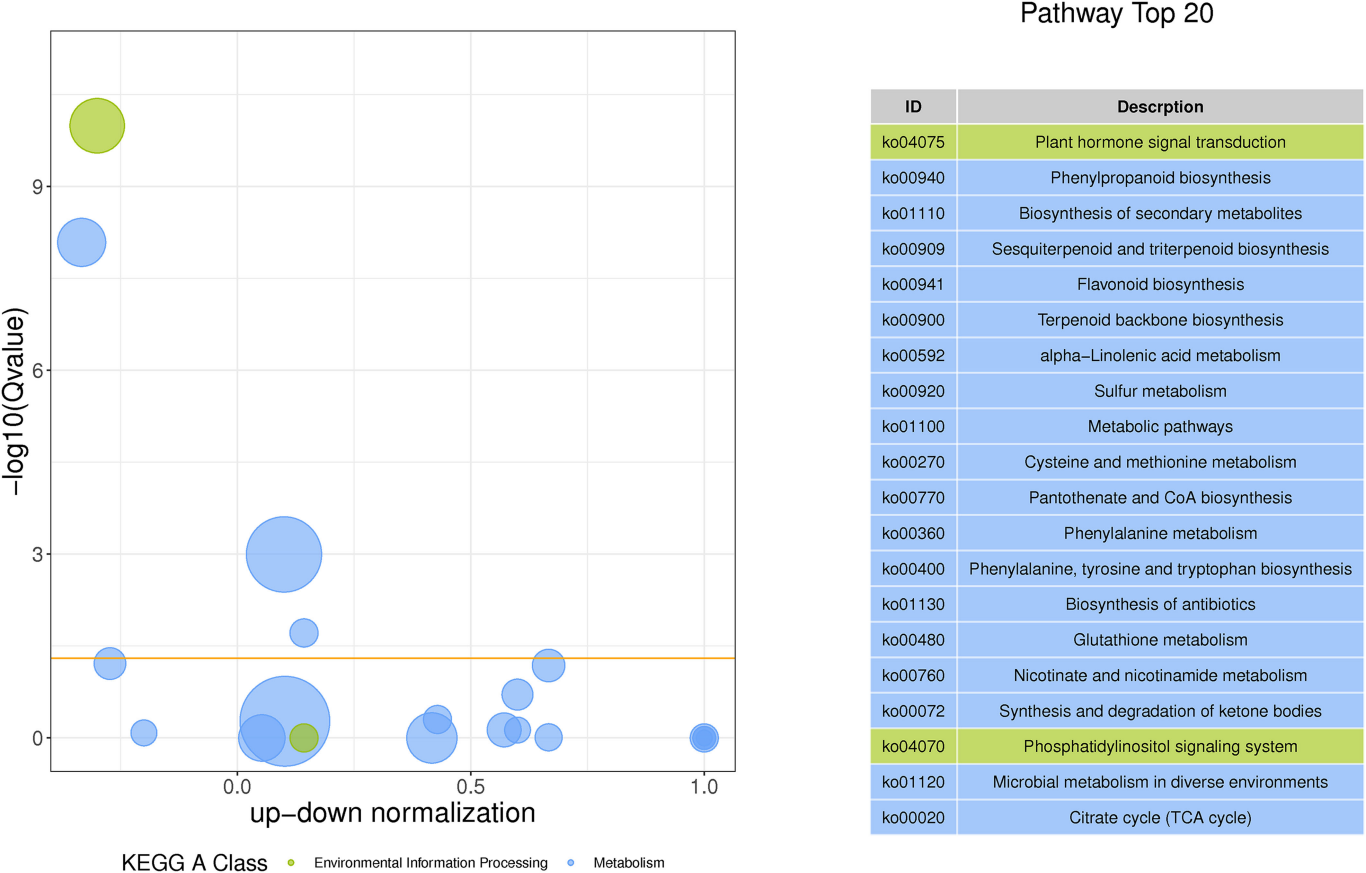
GO/KEGG based on differentially expressed genes shared by WGCNA and the Mfuzz under N vs. T3 treatment groups. The vertical coordinate of the left panel is −log10(*Q*-value), the horizontal coordinate is the *z*-score value (the difference between the number of upregulated differential genes and the number of downregulated differential genes as a proportion of the total number of differential genes), and the yellow line represents the threshold of *Q*-value = 0.05. The list of KEGG terms with the top 20 *Q*-values is shown on the right.

Subsequently, to make a stronger correlation between physiological traits and gene expression patterns and to go for more efficient screening of genes associated with metabolite synthesis, we subjected the genes in C6, MM.black (1,244 DEGs), and MM.magenta (894 DEGs) to Venn plot analyses (**Figure 7A**). Finally, 505 DEGs were targeted. These DEGs were analyzed for KEGG pathway enrichment (**Figure 7B**), and the enriched pathways were “Terpenoid backbone biosynthesis” (11 DEGs), “Phenylpropanoid biosynthesis” (14 DEGs), “Sesquiterpenoid and triterpenoid biosynthesis” (4 DEGs), and “alpha-Linolenic acid metabolism” (6 DEGs), with *P <*0.01 and RichFactor ≥0.05. Also, to understand which pathways played key roles in the continuous MeJA stimulation until the end, we performed KEGG-enrichment analysis for the N and T3 treatment groups. As shown in **Figure 8**, among the four pathways with *Q*-values <0.05, “Phenylpropanoid biosynthesis” and “Sesquiterpenoid and triterpenoid biosynthesis” indicated that both were dominant in the synthesis of plumbagin and saponins.

The results of previous studies showed that “Phenylpropanoid biosynthesis” and “Sesquiterpenoid and triterpenoid biosynthesis” were upstream pathways for the biosynthesis of saponins. “Phenylpropanoid biosynthesis” was an upstream pathway for ketones, and quinones were formed by ketone polyketide reaction formation (Vogt, 2010; Widhalm and Rhodes, 2016; Wang et al., 2019b). Among these pathways, we suggested that Unigene0002103 (*HMGR*), Unigene0028762 (*HMG1*), Unigene0031514 (*FPS2*), Unigene0031515 (*FDS1*), Unigene0003471 (*IPP2*), Unigene0033266 (*F26G*), Unigene0033249 (*4CL1*), Unigene0015669 (*PAL3*), Unigene0033324 (*HST*), etc. might play an important role in the synthesis of saponin and plumbagin.

**Figure 9:**
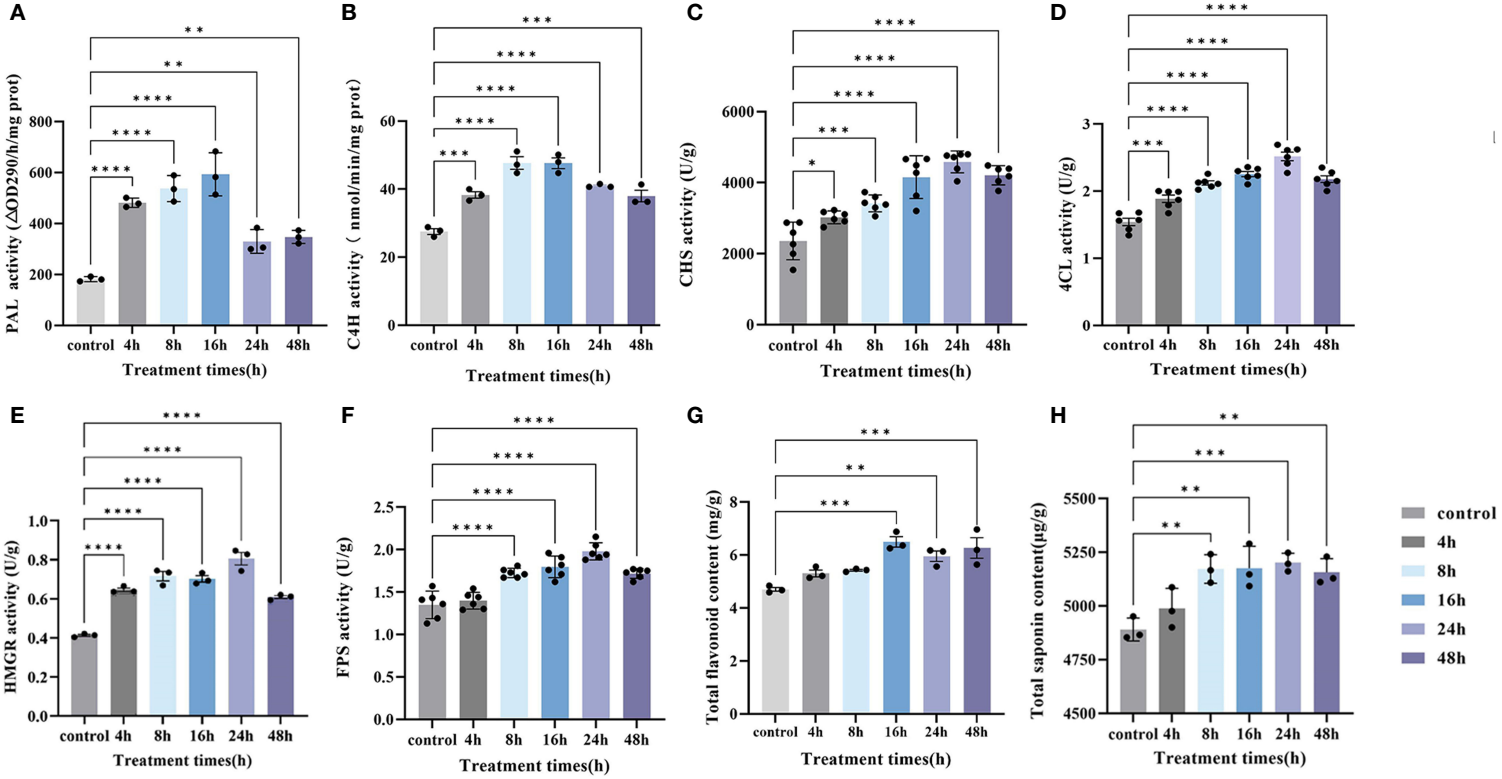
Under MeJA treatment, changed in key synthase activity and substance content over time. **(A)** PAL activity, **(B)** C4H activity, **(C)** CHS activity, **(D)** 4CL activity, **(E)** HMGR activity, **(F)** FPS activity, **(G)** total flavonoid content, and **(H)** total saponin content. Error bars indicate ± SE (*n* = 3 or *n* = 6). Asterisks indicate significant differences between the different groups (*P < 0.05, **P < 0.01, ***P < 0.001, ****P < 0.0001).

Therefore, the contents of total saponins and total flavonoids, as well as PAL, C4H, CHS, 4CL, HMGR, and FPS activities, were examined at four sampling times, and their contents showed a tendency to increase and then decrease or level off with time. Except for the enzyme activities of PAL and C4H and the content of total saponins, which reached the highest value at 16 h, the rest reached the highest peak at 24 h (**Figure 9**). These results were in agreement with the results of previous experiments on the content of plumbagin. We determined the relationship between all the physiological indicators tested and these DEGs (**Supplementary Figure S2**), and the WGCNA and physiological data showed a high degree of positive correlation. Therefore, these genes could efficiently mobilize key reactive enzyme activities in both the saponin and plumbagin synthesis pathways, thereby promoting their co-accumulation.

### 3.5 Analysis of the results of changes in the expression levels of genes related to plumbagin and saponin synthesis

**Figure 10:**
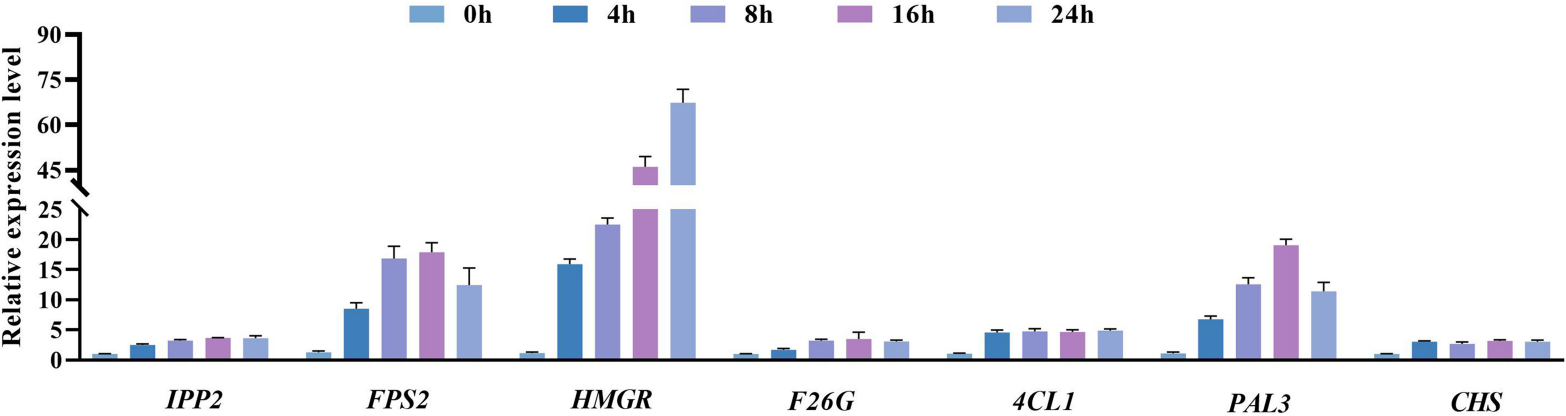
Effect of MeJA on gene expression in the production and synthesis pathways of plumbagin and saponins. Error bars indicate ± SE (*n* = 9).

Eight genes were randomly selected for RT-qPCR analysis to verify the reliability of the RNA-seq results, and it was found that the expression trends of all selected genes were consistent with the RNA-seq results (**Supplementary Figure S3**). We used RT-qPCR to detect key genes that stimulate or regulate the activity of reactive enzymes (**Figure 9**) and verified the transcript expression levels of Unigene0015669 (*PAL3*), Unigene0002103 (*HMGR*), Unigene0033249 (*4CL1*), Unigene0031514 (*FPS2*), Unigene0033266 (*F26G*), Unigene0003471 (*IPP2*), and Unigene0035269 (*CHS2*) in 0 h, 4 h, 8 h, 16 h, and 24 h in hairy roots of PAHR 15834 under MeJA treatment (**Figure 10**). They are considered key genes regulating plumbagin and saponin synthesis. The results showed that *HMGR*, *PAL3*, and *FPS2* showed an increasing and then a decreasing trend based on the expression levels, indicating that they may play a role in the presynthesis stage. Meanwhile, the expression levels of the remaining genes were increasing or increasing first and then leveling off. These results were consistent with those in **Figure 10** and suggested that they might be the key genes that can continuously influence the activity of key enzymes to promote the co-accumulation of plumbagin and saponin.

## 4 Discussion

### 4.1 MeJA plays a vital role in inducing secondary metabolite synthesis and phytohormone signaling

MeJA plays a dual role in plant growth and development, activating plant defense mechanisms (Wang et al., 2021) and acting as an inducer to stimulate the synthesis of secondary metabolites (Chen et al., 2007; Li et al., 2021). In this study, DEGs were abundantly distributed in the “metabolic processes” and “response to stimulus” of the “Biological Processes” and “catalytic activity” in “Molecular Functions” in level 2 GO terms (**Supplementary Figure S4**) in all three modules screened by WGCNA. It indicated that under MeJA treatment, the hairy roots of *P. auriculata* produced a defense response that actively mobilized the accumulation of metabolites as well as the catalytic activity of the cells. This was also proven by Wasternack and Strnad (2019). Notably, in the GO/KEGG enrichment analysis of WGCNA, Mfuzz, and DEGs, we found a significant enrichment in “alpha-linolenic acid metabolism” (**Figure 7B**). This is the JA biosynthesis pathway (Liu et al., 2022), and JA serves as an important signaling hub that regulates an endogenous rise in JA levels as a means to induce the formation of secondary compounds such as terpenoids and flavonoids (Wasternack and Strnad, 2019; Li et al., 2020b). On the other hand, exogenous MeJA can effectively activate response factors in JA signaling, such as *ERFs*, *WRKY*, and *bHLH*, and the spatiotemporal expression patterns of these response factors were consistent with the biosynthesis and accumulation of secondary metabolites. This conclusion has been validated in *Lycoris aurea* (Zhou et al., 2024), and we also found that these transcription factors were actively expressed (**Supplementary Figure S5**). In this study, we hypothesized that the exogenous addition of MeJA could mediate the JA signaling transduction pathway and thus synergistically regulate the increase in the content of secondary metabolites, such as plumbagin, total flavonoids, and saponins. In addition, the studies have shown that genes were upregulated significantly by MeJA treatment related to terpenoid biosynthesis, phenylalanine metabolism, and the JA signaling pathway and the expression of most key enzyme genes were actively regulated in the synthesis pathways of terpenoids and flavonoids (Shi et al., 2023), which was consistent with our research. Wang et al. (2021) suggested that this was a strategy for plants to improve their resistance. Therefore, MeJA acted as a “trauma hormone” and formed “defensive” secondary metabolites in specialized cells, such as the hairy roots in *P. auriculata*, to balance the damage caused by external stimuli, by means of signaling cascades and cellular tissue accumulation (Talebi et al., 2018). Plants competed to form these special secondary metabolites in order to adapt and maintain metabolic balance (Garrido et al., 2022), and this was responsible for the increase in plumbagin content, total flavonoids, and saponins (**Figures 2C**, **9G, H**).

### 4.2 Phenylpropanoid biosynthesis and terpenoid backbone biosynthesis played key roles in the synthesis of plumbagin and saponins

Plants synthesize phenylpropanoid compounds, phenylpropanoid derivatives, terpenoids, etc. in response to this specific environmental change in order to manage and balance the effects of the environment in which they are growing and/or other life forms (Le Roy et al., 2016; Dong and Lin, 2021). Phenylpropanoid biosynthesis and terpenoid backbone biosynthesis are major pathways for the production of a variety of specialized metabolites to withstand environmental stresses and complete growth and development (Vogt, 2010; Tholl, 2015; Dong and Lin, 2021). The phenylpropane pathway could be regulated by MeJA application, mainly through the activation of PAL, C4H, 4CL, etc., and their enzymatic activities were enhanced by the expression of key genes involved in phenylpropane metabolism, promoting key biosynthetic reactions (Zhang et al., 2024a). In our study, a large number of transcription factors such as *MYBs*, *WRKYs*, and *ERFs* were detected in the three modules of WGCNA (**Supplementary Figure S5**), which were key to the activation of the phenylpropanoid pathway (Bonawitz et al., 2014; Wang et al., 2019a; Xiao et al., 2023). The production of multiple secondary metabolites was enhanced by these transcription factors through transcriptional reprogramming, which activated downstream genes that control reactive enzymes during this process (Moghadam et al., 2024), such as the activation of the first enzymatic reaction. These reactions were as follows: PAL catalyzed the conversion of phenylalanine to cinnamic acid, which is subsequently hydrolyzed by C4H and converted to p-coumaric acid; under the action of 4CL, p-coumaric acid formed coumaroyl CoA, which then binds to CHS to form the ketone backbone (Le Roy et al., 2016; He et al., 2022). In early studies, plumbagin was characterized as the key and was specific to type III polyketide synthases (PKS) in the synthetic pathway, in which CHS was noted to be a type III PKS that was ubiquitous in plants and was involved in polyketone (Jadhav et al., 2014). In RNA-seq, high expression of *CHS* genes and a large increase in CHS enzyme activity led to the accumulation of plumbagin in the hairy roots of *P. auriculata.* Therefore, the accumulation of daidzein in the hairy roots of *P. auriculata* might be mainly influenced by the CHS in “phenylpropanoid synthesis.” Fortunately, in addition to the above enzymes, we also found other enzymes, such as caffeic acid 3-*O*-methyltransferase (Unigene0025020), shikimate *O*-hydroxycinnamoyl transferase (Unigene0033324), and cinnamyl-alcohol dehydrogenase (Unigene0017777), in the “phenylpropanoid biosynthesis” pathway that we eventually focused on. These enzymes have been shown in existing studies to catalyze the production of aldehydes and to provide precursors for the formation of the benzene and quinone rings of quinones (Widhalm and Rhodes, 2016; Dong and Lin, 2021). Therefore, we suggested that MeJA treatment in the hairy roots of *P. auriculata* activates the phenylpropanoid biosynthesis pathway to promote quinone accumulation. Unfortunately, the metabolic network of plants is complex and diverse, as is the entire process of polyketone, which goes through aldehyde condensation, aldehyde cyclization, hydroxylation, and oxidation (Jindaprasert et al., 2008), which was due to the fact that we did not have a complete understanding of the synthetic pathway and did not validate the function of key genes in forming ketones or aldehydes of plumbagin, which we will complete in future studies.

On the other hand, terpenoid synthesis consists of two parts: terpenoid backbone biosynthesis and backbone modification (Tholl, 2015). In the b terpenoid backbone biosynthesis, IPP and dimethylallyl pyrophosphate (DMAPP) were cyclized or aligned to form different types of oligomers, followed by the assembly of a series of terpene skeletons catalyzed by various cyclases (Zhang et al., 2023). It has been shown in terpenoid backbone biosynthesis that they are synthesized via the isoprenoid pathway. The active intermediate IPP and the MVA pathway, which were in the mitochondria and cytoplasm, were realized in the presence of HMGR, FPS, etc (Xu et al., 2018). Among them, IPP was also a key precursor substance for the synthesis of steroid saponins (Li et al., 2023b). After RNA-seq analysis by WGCNA and Mfuzz, 11 DEGs were finally focused on the “terpenoid backbone biosynthesis” pathway (**Figures 6D**, **7B**), among which two genes related to HMGR and four genes related to FPS were upregulated. In the RT-qPCR expression level analysis of one gene related to HMGR and FPS as well as the change in the activities of HMGR and FPS at each treatment time (**Figure 9**), this indicated that MeJA treatment promoted the accumulation of triterpenoid saponins. In previous studies, alpha-linolenic acid metabolism and terpenoid backbone biosynthesis pathways were proposed, which were crucial for MeJA activation of terpenoid biosynthesis, respectively (Zhang et al., 2024b). These results have been similarly validated in our study (**Figure 7**). In addition, the high expression of *F26G* (Unigene0033266) (**Supplementary Figures S2**, **10**), a synthase gene that produces steroidal saponins, was also found in our results, so we believed that “terpenoid backbone biosynthesis” was the key to the synthesis of saponins.

### 4.3 Plumbagin and saponin biosynthesis shared the MVA pathway and activated the PAL and HMGR “gateway enzymes” for co-accumulation

The plant shikimic acid pathway is the upstream pathway for phenylpropanoid biosynthesis (Vogt, 2010), and phenylalanine is the end product of the shikimic acid pathway and the initiator of the MVA pathway (Tholl, 2015; Zhang and Liu, 2015). The MVA pathway serves as a core metabolic pathway that provides precursors for the cytoplasmic biosynthesis of ketones, aldehydes, and triterpenoids, as well as for the biosynthesis of terpenoids (e.g., ubiquinone, polyphenol) in the mitochondria (Tholl, 2015). PAL, the gateway enzyme for phenyl propionic acid metabolism, constructs an upstream reaction network for flavonoids (Liu et al., 2023) and realizes the shift of the shikimic acid pathway to the various branches of phenyl propionic acid metabolism by catalyzing the generation of trans-cinnamic acid from phenylalanine (Zhang and Liu, 2015; Dong and Lin, 2021), thus affecting the changes in key genes involved in the initiation of phenylpropanoid (*C4H* and *4CL*) and flavonoid (*CHS* and *CHI*) biosynthesis (Wan et al., 2023; Zeng et al., 2024). HMGR is the major rate-limiting enzyme regulating isoprenoid biosynthesis (Nieto et al., 2009), and it catalyzes the irreversible conversion of 3-hydroxy-3-methylglutaryl CoA (HMG CoA) to mevalonate, which is synthesized through the MVA pathway in saponins (Joga et al., 2024). A study by Yin et al. (2023) showed that optimizing its gene surface enzyme activity could promote the synthesis of downstream substances, such as efficiently controlling the accumulation of terpenoids (Lu et al., 2022; Zhang et al., 2023). Unlike previous studies, we found that MeJA activated the two “gateway enzymes” PAL and HMGR after treating the hairy roots in *P. auriculata*, and the expression levels of *PAL3* and *HMGR*, which were involved in the regulation of the enzymes, showed a tendency of increasing and then decreasing with the increase in treatment time, which was consistent with the accumulation of plumbagin and saponins. In addition, precursor substances involved in the MVA pathway required induction from JA. In the study, we also found the involvement of transcription factor families such as *ERFs* and *bHLHs* (**Supplementary Figure S5**), suggesting that JA-mediated and JA-regulated genes were involved in genes regulating the formation of secondary metabolic compounds (Major et al., 2017). Based on this, we concluded that MeJA treatment took the lead in affecting the JA signaling and the shikimic acid pathways in *P. auriculata*. Subsequently, the high expression of *PAL3* and *HMGR* in the MVA pathway was stimulated and induced by the high expression of *CHSs* downstream of the plumbagin synthesis pathway and *IPPs* and *FPSs* in the saponin synthesis pathway, which activated the synthetic reactive enzymes to synthesize plumbagin and saponins methodically under the shared metabolic flux of the MVA pathway.

### 4.4 Soluble sugars and soluble proteins were important precursor metabolites affecting the synthesis of plumbagin and saponins

The primary metabolites of plants, such as sugars and proteins, are plant growth regulators and secondary metabolite precursors, and their content levels are affected, such as during plant resistance to environmental changes, secondary metabolism synthesis, and so on (Murcia et al., 2017; Li et al., 2024). In the MM.cyan module, we found Unigene0032085 (*At5g47720*), which is annotated as acetyl-CoA C-acetyltransferase. It is formed by the condensation of acetyl-CoA, an important starting molecule for metabolite biosynthesis, and this type of enzyme is produced in large quantities in gluconeogenesis (Vishwakarma et al., 2013). Previous studies reported that three acetyl-CoA were converted to IPP in the MVA pathway, which is a precursor for saponin synthesis (Chen et al., 2017). In this study, the soluble sugar content in the hairy roots of *P. auriculata* increased substantially, providing a large amount of acetyl-CoA for the subsequent synthesis of secondary metabolites. It was hypothesized to possibly provide energy reserves and a synthetic environment for the completion of secondary metabolisms, such as saponins, and to lay the foundation for the activation of the downstream enzyme-linked reactions to achieve biosynthesis. On the other hand, the soluble proteins play a role in plant metabolism by recognizing signals and transporting them from the endoplasmic reticulum (ER) to various locations involved in biological reactions (Hadlington and Denecke, 2000). In MM.cyan, there was a large enrichment of DEGs in “Protein processing in endoplasmic reticulum” and “protein export” (**Figure 6B**). In the MVA pathway, enzymes are mostly located on the cytoplasmic lysosome or endoplasmic reticulum, and precursor-reactive enzymes for the synthesis of plumbagin and saponin have been shown to act on the endoplasmic reticulum through transmembrane regions, such as HMGR (Tholl, 2015). In addition, amino acids are important energy metabolites and precursors of bioactive molecules and play a key role in the saponin synthesis pathway (Wang et al., 2019b). The higher glycolysis and serine levels, which were induced under abiotic stress, trigger the MVA pathway to promote saponin biosynthesis. Therefore, we hypothesized that the increase in soluble proteins in the hairy roots of *P. auriculata*, in addition to responding to MeJA, might also be actively engaged in transmembrane transport and energy transfer, and the synthesis of reactive enzymes would be allowed to function in a suitable environment.

## 5 Conclusion

**Figure 11:**
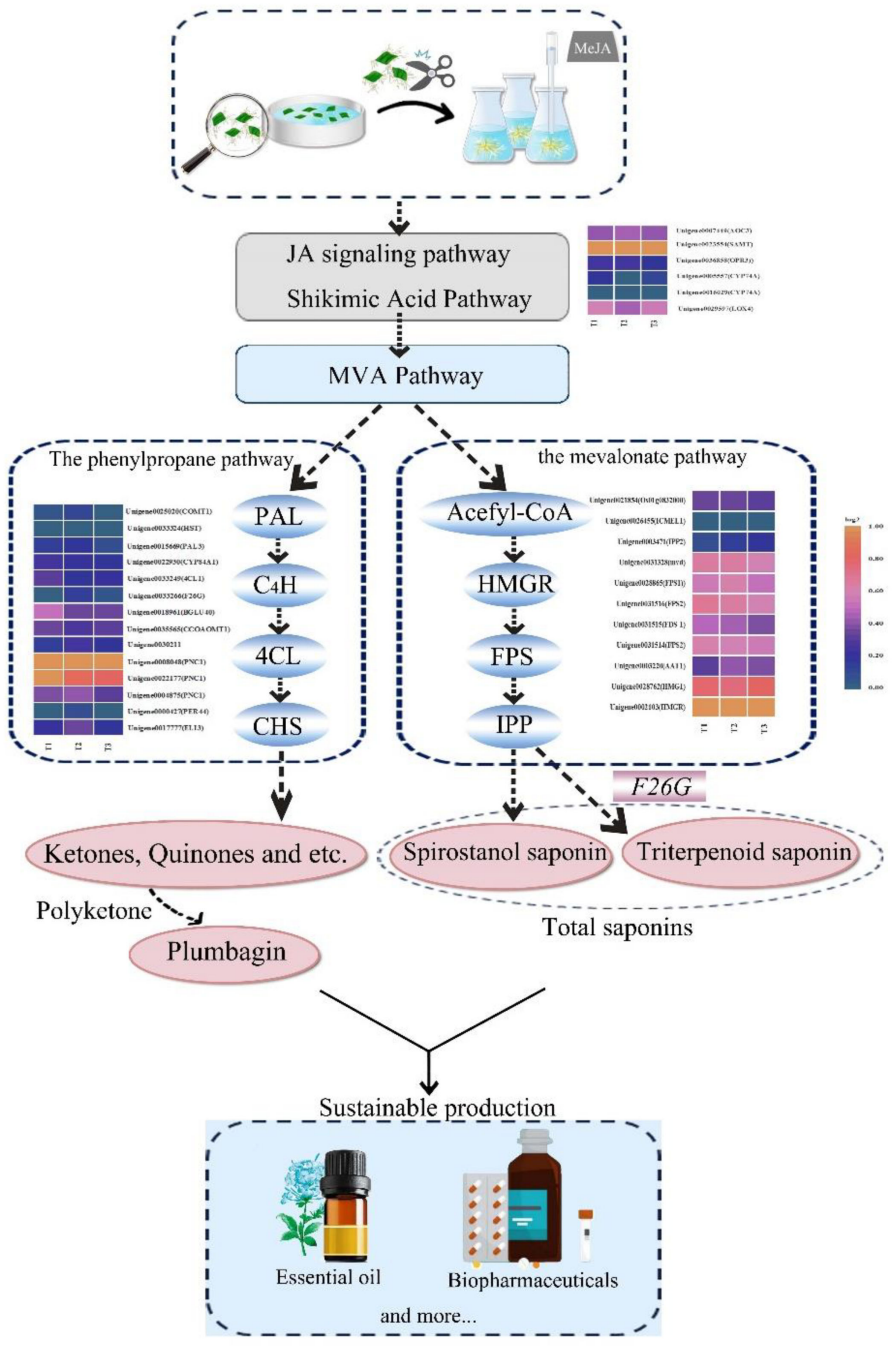
Prediction of the synthesis pathway in plumbagin and saponins after MeJA treatment of the hairy roots of *Plumbago auriculata*.

The materials used in this study were the hairy roots of *P. auriculata.* A significant accumulation of plumbagin and saponin in hairy roots was found after treating PAHR 15834 with 100 μmol/L of MeJA. Combining RNA-seq profiling and analysis using Mfuzz and WGCNA, the pathways for their synthetic accumulation were predicted, as shown in **Figure 11**. We hypothesize that MeJA could effectively stimulate the JA signaling and shikimic acid pathways and mediate the MVA pathway. Meanwhile, the energy and synthetic environments provided many soluble sugars and soluble proteins for the formation of plumbagin and saponins. Under the shared metabolic flux of this pathway, it mediated the high expression of *PAL3* and *HMGR*, successfully activating the activities of the “gateway enzymes” PAL and HMGR in the phenylpropane and mevalonate pathways, and induced the high expression of *CHSs* in the downstream plumbagin synthesis pathway, *FPSs* and *IPPs* in the saponin synthesis pathway, and *CHSs* in the downstream plumbagin synthesis pathway. Among them, DEGs were heavily enriched in phenylpropanoid biosynthesis and terpenoid backbone biosynthesis, and it was hypothesized that the expression of DEGs in the pathway activated the activity of a series of reaction synthases, which formed a large number of ketones and aldehydes under the action of their synthesis reaction enzymes and ultimately contributed to the co-accumulation of plumbagin and saponins through the highly efficient polyketone and a high level of sugar metabolism and amino acid levels. The study provides a theoretical basis for carrying out factory refinement and biosynthesis of plumbagin and saponin and also provides a new idea for the development and application of multifunctional flower crops.

## Data availability statement

The original contributions presented in the study are included in the article/**Supplementary Material**. The original data and analysis file in this study can be found in Science DB (https://www.scidb.cn/en/s/bqA3Mj).

## Author contributions

YL: Formal analysis, Methodology, Software, Writing – original draft, Writing – review & editing. Z-aZ: Formal analysis, Methodology, Software, Writing – original draft. JH: Formal analysis, Investigation, Writing – original draft. TL: Conceptualization, Investigation, Resources, Writing – review & editing. QC: Supervision, Validation, Resources, Writing – review & editing. JL: Investigation, Project administration, Supervision, Writing – review & editing. LY: Project administration, Investigation, Supervision, Writing – review & editing. DH: Investigation, Project administration, Resources, Writing – review & editing. SG: Data curation, Methodology, Project administration, Resources, Writing – review & editing.
