# Supplementary material for: MeJA-induced hairy roots in Plumbago auriculata L. by RNA-seq profiling and key synthase provided new insights into the sustainable production of plumbagin and saponins
Source: Front Plant Sci. 2024 Jul 12;15:1411963. doi: 10.3389/fpls.2024.1411963 (PMC11272555; doi:10.3389/fpls.2024.1411963)
Supplement: Supplementary file 1 [file DataSheet_1.docx]

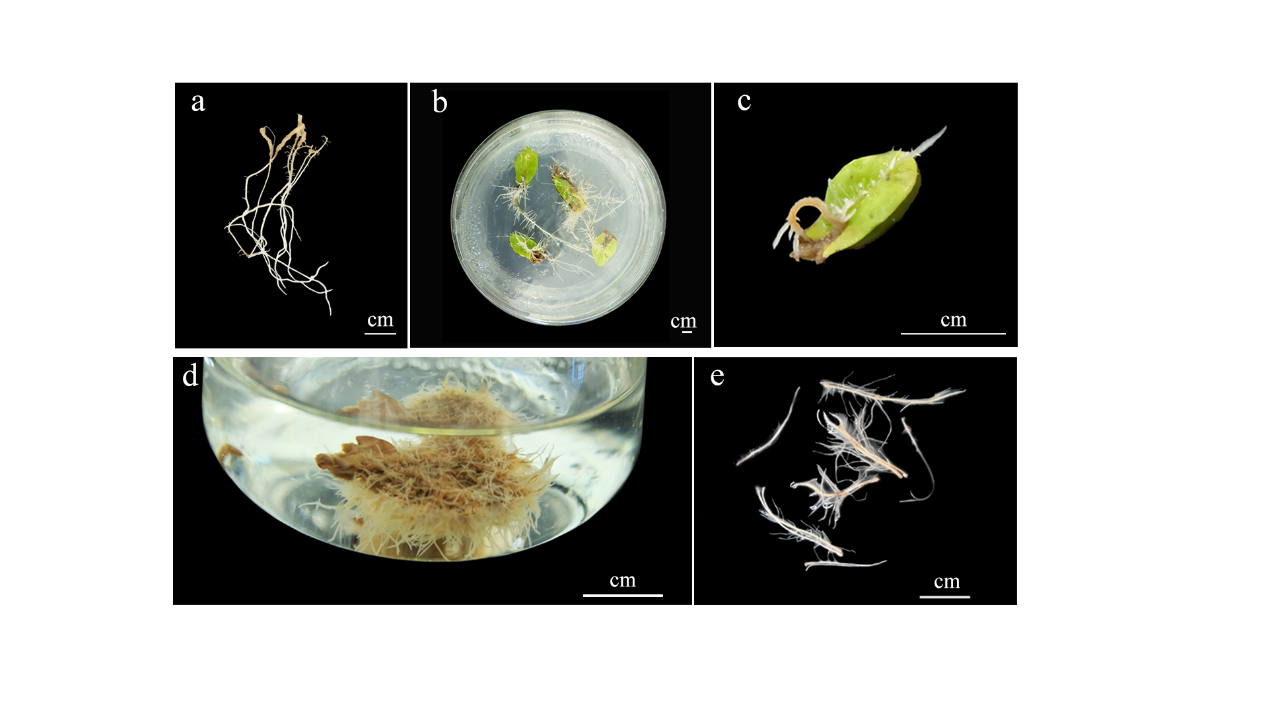


**Figure S1.** Morphological diagrams of PAHR 15834. a) the root system of the histocultured seedling in *P. auriculata.*; b) the induced culture in PAHR 15834 hairy roots medium; c) the initial morphology of the induced hairy roots in PAHR 15834; d) the monoclonal clone in PAHR 15834; e) the isolated hairy roots in PAHR 15834.


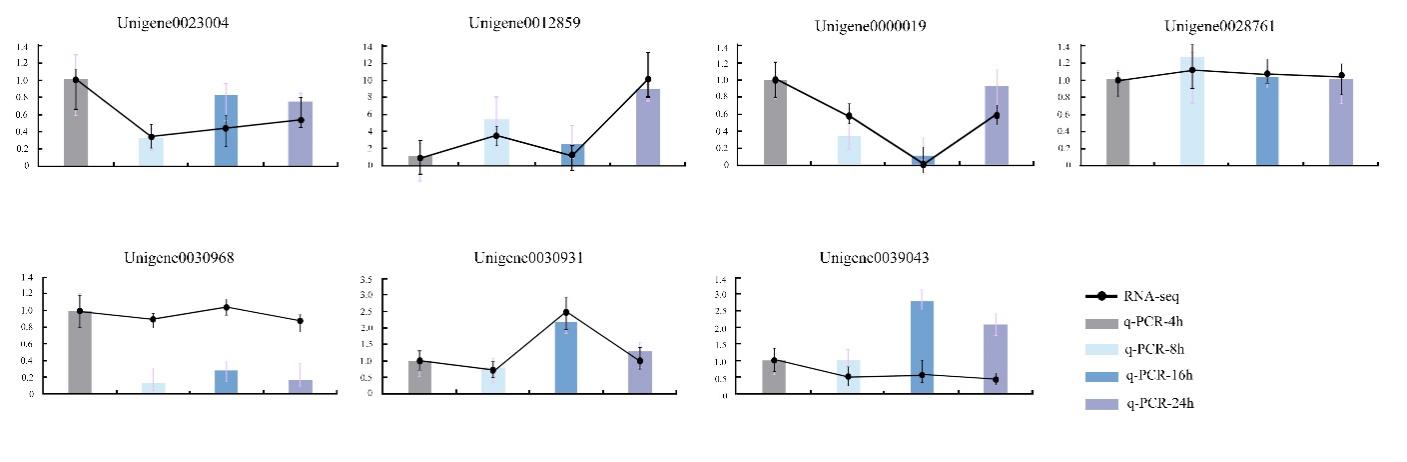


**Figure S2.** The RNA-seq and RT-qPCR expression trend plots of 8 genes. Error bars indicate ± SE (n = 9).


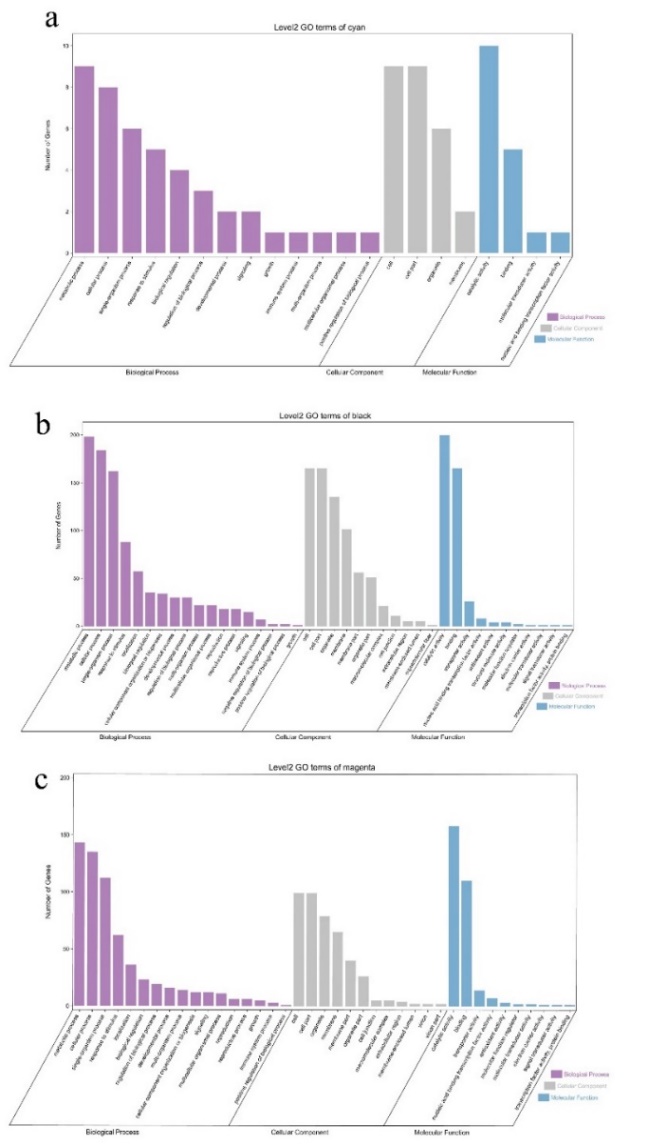


**Figure S3** GO enrichment analysis based on WGCNA, physiological parameters, and DEGs. a) GO enrichment pathway of DEGs in MM.cyan; b) the GO enrichment pathway of DEGs in MM.black; c) the GO enrichment pathway of DEGs in MM.magenta.


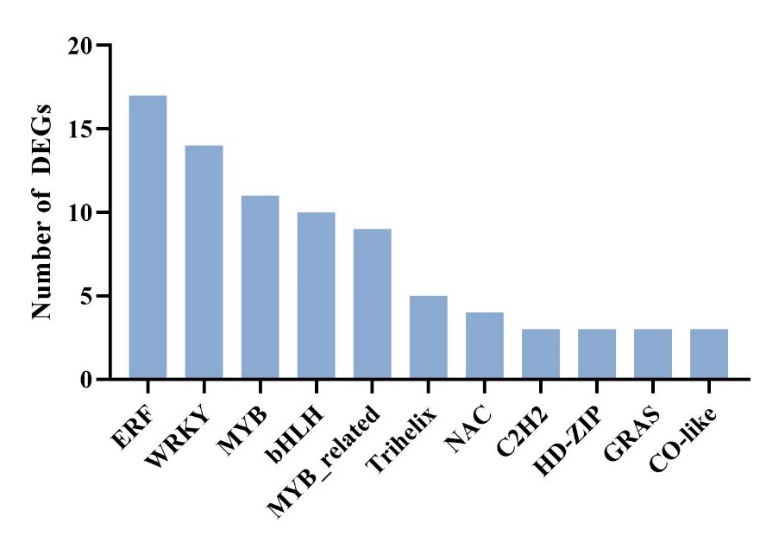


**Figure S4**. Top 10 statistics for number of transcription factors in 3 modules of WGCNA

**Table S1** RT-qPCR Primer list

| NO. | Name | Primers |
| --- | --- | --- |
| 1 | ACTIN-F | CTGGAATTGCTGACCGTATGAG |
|  | ACTIN-R | GCCAAGATGGATCCTCCAATC |
| 2 | Unigene0023004-F | TAACTGGAAAGGAACTGGAGC |
|  | Unigene0023004-R | CGGAAGGCAACCAAGTTTC |
| 3 | Unigene0012859-F | GCCGCAGATGATAGAGGACAA |
|  | Unigene0012859-R | GGACAGCCCAGAACACCTT |
| 4 | Unigene0000019-F | TTGTCGCCTCCTTCACTCTCG |
|  | Unigene0000019-R | CGTCGCCTTCCTCCTCCAT |
| 5 | Unigene0030968-F | GCTTAGCATACAGTCTCACA |
|  | Unigene0030968-R | GGAAGAACTTGCCTGTGT |
| 6 | Unigene0028761-F | AGGTGAAGGTGTGGCTTGG |
|  | Unigene0028761-R | GGTTGGGAATCTTGGTGACAGT |
| 7 | Unigene0030931-F | GCAGCACATAAATGAGGAACAGAA |
|  | Unigene0030931-R | GCTCGGTTGATTGGTTGAAGAC |
| 8 | Unigene0039043-F | AAAGGCGTGGGCTGAAGGT |
|  | Unigene0039043-R | GCCTCCTCCACAGCAGTATCAA |
| 9 | Unigene0035266-F | CGGAATCAGCGACTGGAACTCAC |
|  | Unigene0035266-R | CATTCAACACCTGCCTCGTAGCC |
| 10 | Unigene0015669-F | CAACAACTCTGCCACTAC |
|  | Unigene0015669-R | CTCCCTGACGAACCTAT |
| 11 | Unigene0002103-F | AGCAATCGTCCAAAGT |
|  | Unigene0002103-R | GCGTCAGATGCCCATA |
| 12 | Unigene0033249-F | ACAGAGCCCAACCCAGAAGAG |
|  | Unigene0033249-R | AGCCTAAACCACGGAGACAGC |
| 13 | Unigene0031514-F | CATAGCCTTCACCACCAGCCAAG |
|  | Unigene0031514-R | TCTTCCGCTCGGTAGGATGACTTC |
| 14 | Unigene0033249-F | CACCAACAGTAGTACCAACCACAAC |
|  | Unigene0033249-R | GGCAGCGGAGATAGGGAAACAG |
| 15 | Unigene0033266-F | AGAGTTTGGCGACAGG |
|  | Unigene0033266-R | TTGACAGCGGCAGCAT |
| 16 | Unigene0003471-F | AGGACAGAGCCCAACCCAGAAG |
|  | Unigene0003471-R | TAAGCCCATCCTCTCCAGCATCC |
| 17 | Unigene0035269-F | CGGAATCAGCGACTGGAACTCAC |
|  | Unigene0035269-R | CATTCAACACCTGCCTCGTAGCC |

**Table S2.** Summary of the *P. auriculata* transcriptome sequencing data

| Sample | Clean reads | GC content(%) | ≥Q30(%) | Mapped ratio(%) |
| --- | --- | --- | --- | --- |
| N-1 | 41,212,236 | 47.11 | 93.49 | 86.56 |
| N-2 | 45,690,068 | 47.12 | 93.09 | 86.50 |
| N-3 | 41,193,358 | 46.91 | 92.72 | 86.39 |
| T1-1 | 39,141,222 | 47.14 | 92.81 | 86.17 |
| T1-2 | 41,194,672 | 47.02 | 92.91 | 86.42 |
| T1-3 | 45,691,156 | 48.27 | 93.36 | 86.92 |
| T2-1 | 49,851,410 | 46.84 | 93.37 | 86.20 |
| T2-2 | 47,638,854 | 46.95 | 93.60 | 86.25 |
| T2-3 | 47,308,988 | 46.78 | 93.85 | 86.03 |
| T3-1 | 45,477,320 | 46.97 | 93.38 | 86.34 |
| T3-2 | 41,949078 | 50.02 | 92.57 | 86.02 |
| T3-3 | 42,542204 | 46.88 | 93.58 | 86.27 |
